# Voxel-based morphometric brain comparison between healthy subjects and major depressive disorder patients in Japanese with the s/s genotype of 5-HTTLPR

**DOI:** 10.1038/s41598-017-04347-8

**Published:** 2017-06-21

**Authors:** Natsuki Igata, Shingo Kakeda, Keita Watanabe, Satoru Ide, Taro Kishi, Osamu Abe, Ryouhei Igata, Asuka Katsuki, Nakao Iwata, Reiji Yoshimura, Yukunori Korogi

**Affiliations:** 1 Department of Radiology, University of Occupational and Environmental Health, Kitakyushu, Japan; 2 Department of Psychiatry, University of Occupational and Environmental Health, Kitakyushu, Japan; 3 Department of Psychiatry, Fujita Health University, School of Medicine, Toyoake, Japan; 4 Department of Radiology, Graduate School of Medicine, University of Tokyo, Tokyo, Japan

## Abstract

Individuals with s/s genotype of serotonin transporter gene-linked promotor region (5-HTTLPR), which appear with a high frequency in Japanese, exhibit more diagnosable depression in relation to stressful life events than those with the s/l or l/l genotype. We prospectively investigated the brain volume changes in first-episode and medication naïve major depression disorder patients (MDD) with the s/s genotype in Japanese. We assessed the differences between 27 MDD with the s/s genotype and 44 healthy subjects (HS) with the same genotype using a whole-brain voxel-by-voxel statistical analysis of MRI. Gray matter volume in a brain region with significant clusters obtained via voxel-based morphometry analysis were measured and, as an exploratory analysis, evaluated for relationships to the subcategory scores (core, sleep, activity, psychic, somatic anxiety, delusion) of the Hamilton Depression Rating Scale (HAM-D) and the Social Readjustment Rating Scale (SRRS). The brain volume in the left insula lobe was significantly smaller in the MDD than in the HS. The left insula lobe volume correlated negatively with the “psychic” score of HAM-D and the SRRS. In a Japanese population with the s/s genotype, we found an atrophy of the insula in the MDD, which might be associated with “psychic” symptom and stress events.

## Introduction

One of the major genetic factors determining inter-individual differences in stress reactivity is the serotonin transporter (5-HTT) gene polymorphism, which mediates reuptake and recycling of released serotonin following neuronal stimulation. Neurotransmission mediated by serotonin (5-HT) contributes to many physiological functions, such as motor activity, food intake, sleep, and emotional states^1, 2^. There are two common alleles: a 44-bp insertion (l*-*allele) or deletion (s*-*allele). Transcriptional activity of the l-allele of the gene is twice that of the s-allele^3^. Thus, the s promoter allelic variant is linked to reduced 5-HTT mRNA expression, resulting in less serotonin reuptake than with the l-allelic variant^4, 5^.

Many studies have suggested an association between the 5-HTT genotype and physiological reactivity to acute stressors^6, 7^. Gotlib *et al*. found that individuals with the s/s genotype showed enhanced cortisol secretion in response to acute psychological stressors^7^. The most recent meta-analysis regarding the 5-HTT gene-linked polymorphic region (5-HTTLPR) confirmed the robust link between the s/s genotype of the 5-HTTLPR, the experience of stress, and resultant depression^8^. Therefore, individuals with the s/s genotype are more sensitive to the depressogenic effects of all stressful life events than those with the s/l or l/l genotype.

For the 5-HTT genotype, the distribution of alleles in Caucasians is significantly different from that in the Japanese population. Lesch *et al*. reported that a PCR-based genotype analysis in Caucasian subjects revealed allele frequencies of 32% for the l/l genotype, 49% for the l/s, and 19% for the s/s^4^. In contrast, in 501 Japanese healthy subjects, the frequency distribution of the l/l, l/s, and s/s genotypes were 3% (n = 16), 32% (n = 159), and 65% (n = 326), respectively^9^. Therefore, because differences in racial genetic backgrounds may be contributing factors, additional assessments regarding the 5-HTT genotype must be conducted separately for each race group.

Major Depressive Disorder (MDD) is not a homogeneous disease. For example, Parker has proposed that depression may have various forms^10^, and Ostergaard and colleagues^11^ pointed out that MDD may present in 1497 different combinations of the symptoms that are used to define it^12^. Recent data which demonstrate the different neurobiological underpinnings, pathways and required treatments for at least four different “subtypes” of depression based upon separation of MDD symptoms into distinct categories according to symptom coherence^13, 14^. In other words, MDD is a heterogeneous disorder, which origins from various pathophysiology. We focused MDD dimensions according to the components proposed by Seretti *et al*.^15^, in which Hamilton Depression Rating Scale (HAM-D) items were grouped into 6 factors (core, sleep, activity, psychic, somatic anxiety, and delusion).

Morphological brain abnormalities in MDD patients may be attributable to genetic- and epigenetic factors that regulate brain development and neurodegeneration. In MDD, many previous magnetic resonance imaging (MRI) studies using the voxel-based morphometry (VBM) technique have reported evidence of a relationship between brain volume and genetic factors, including brain-derived neurotrophic factor (BDNF), norepinephrine transporter (NET) gene (SLC6A2), and methylenetetrahydrofolate reductase (MTHFR)/catechol-O-methyltransferase (COMT) polymorphisms^16–22^. To our knowledge, however, there has only been one VBM study of MDD patients and HS regarding the association between the triallelic polymorphism in 5-HTTLPR and gray matter (GM) brain volume. The authors assessed the effect of 5-HTTLPR polymorphisms by comparing l-allele individuals versus s-allele ones (the l/s genotype as well as the s/s genotype) because of the limited number of s/s genotype individuals available. Thus, the morphological brain changes in MDD patients with the s/s genotype of 5-HTTLPR have not been fully evaluated. We therefore examined the brain volume changes in first-episode and medication naïve MDD patients with the s/s genotype of 5-HTTLPR using 3-Tesla MRI data from a Japanese population.

## Materials and Methods

### Study Participants

Human experiments were carried out in accordance to guidelines provided and approved by the Institutional Review Board of University of Occupational and Environmental Health School of Medicine, Japan (approval number: H25-13). The protocol of this prospective study was approved by the Ethics Committee of the University of Occupational and Environmental Health. All of the participants provided their written informed consent to participate in the study. Twenty-seven first-episode treatment-naïve MDD patients were recruited. All patients were consistent right-handers. A majority of subjects in this study participated in an earlier published study, which analyzed the relationship of brain volume to the catechol-*O*-methyl transferase (COMT)^21^, brain-derived neurotrophic factor (BDNF)^19^, and norepinephrine transporter (NET)^22^ gene polymorphism. A psychiatrist (K.H. with seven years of experience in psychiatry) diagnosed a major depressive episode using the Structured Clinical Interview according to the Diagnostic and Statistical Manual of Mental Disorders (DSM)-IV-TR criteria. The severity of depression was evaluated using the17-item HAM-D. The exclusion criteria were a history of neurological diseases or other physical diseases and the presence of comorbidities with other disorders (no evidence of schizoaffective disorder, bipolar disorder, Axis II, personality disorders, or mental retardation).

Forty-seven healthy subjects (HS) were also recruited via an interview conducted by the same psychiatrist using the Structured Clinical Interview for DSM-IV, non patient edition. None of them had a history of serious medical or neuropsychiatric illness or a family history of major psychiatric or neurological illness among their first-degree relatives.

### Genotyping

Seventy-four subjects from the neuroimaging study provided a blood sample, from which DNA was extracted using standard laboratory protocols. DNA was isolated from peripheral blood mononuclearcells using the QIAamp DNA Mini-Kit (QIAGEN, Tokyo, Japan). Genotyping was carried out with a polymerase chain reaction (PCR) SNP genotyping system using the BigDye Terminator v3.1 Cycle Sequencing Kit (Life Technologies Japan, Tokyo, Japan). The DNA was read using a BMG Applied Biosystem 3730xI DNA Analyzer (Life Technologies Japan). We used a forward primer (5′-GGC GTT GCC GCT CTG AAT GC-3′) and a reverse primer (5′-GAG GGA CTG AGC TGG ACA ACC AC-3′) for the 5-HTTLPR polymorphism. The genotyping revealed that all 27 MDD patients had the s/s genotype of 5-HTTLPR. Among 47 HS, 3 had l/s, and 44 had s/s genotypes of 5-HTTLPR. Therefore, the 44 HS with the s/s genotype of 5-HTTLPR were assessed in this study.

### MRI and Image Processing for Voxel-Based Morphometry (VBM)

All MDD patients underwent brain MRI before receiving antidepressant medication or psychotherapy. Therefore, all participants were medication naïve at the time of MRI. MRI data were obtained using a 3.0-Tesla scanner (Signa EXCITE 3 T; GE Medical Systems, Milwaukee, WI, USA) with a 3-dimensional fast-spoiled gradient-recalled acquisition with steady state (3D-FSPGR). The following parameters were used: repetition time ms/echo time ms/inversion time, 10/4.1/700; flip angle, 10; field of view, 24 cm; section thickness, 1.2 mm; and resolution, 0.9 × 0.9 × 1.2 mm. All of the images were corrected for image distortion due to gradient non-linearity using “GradWarp”^23^ and for intensity inhomogeneity using “N3”^24^. The SPM8 software program was used for the image processing of VBM.

The 3D-FSPGR images in native spaces were spatially normalized and segmented into GM, white matter, and cerebrospinal fluid images, and were intensity-1modulated using the Diffeomorphic Anatomical Registration Through Exponential Lie Algebra (DARTEL) toolbox in SPM8^25^. DARTEL was proposed by Ashburner as an alternative method for normalization in the SPM package^26^. For intensity modulation, the voxel values of the segmented images were multiplied by the measure of the warped and unwarped structures derived from the nonlinear step of the spatial normalization. This step converted the relative regional GM density into absolute GM density, expressed as the amount of GM per unit volume of brain tissue before spatial normalization. The resulting modulated GM and white matter images were smoothed with an 8-mm Gaussian kernel.

### Hamilton Depression Rating Scale and Social Readjustment Rating Scale

To evaluate the specific subcategories of depressive symptoms, the HAM-D items were grouped according to the following factors: core (items 1, 2, 7, 8, 10, and 13), sleep (items 4.6), activity (items 7 and 8), psychic (items 9 and 10), somatic anxiety (items 11.13), and delusion (items 2, 15, and 20), as described by Serretti *et al*.^15^. In addition, stressful life events were assessed using the Social Readjustment Rating Scale (SRRS), which is a major life events inventory developed by Holmes and Rahe^27^. The SRRS assesses frequency of common stressful life events that occurred over the previous 12 months. The SRRS has been widely used in studies of psychosocial stress and illness, and includes 43 stressful life events, each scored from 0 to 100 units of life change (ULC).

### Statistical Analysis

For the analysis of demographic and clinical characteristics of participants, a multivariate analysis of variance was performed to compare the differences in age and total GM volume between HS and MDD patients. In the VBM analysis, statistical analyses were performed using the SPM8 software program. The morphological changes in the GM were assessed using a two-sample t-test with diagnosis status. Age, sex, and total GM volume were included as covariates of no interest into all analyses with HS as confounding variables. The differences in the GM volume between MDD patients and HS with the s/s genotype were assessed at the whole-brain level. This analysis yielded statistical parametric maps (SPMs [t]) based on a voxel-level height threshold of p < 0.001. We used cluster level family wise error (FWE) correction. The significance level was set at an FWE-corrected p < 0.05. GM volume in a brain region with significant clusters obtained on the VBM analysis were measured by using region of interest (ROI) analyses and, as an exploratory analysis, evaluated for relationships to the subcategory scores of HAM-D and the SRRS using Spearman’s rank correlation.

The statistical analyses were performed using the statistical software package StatView 5.0 (SAS Institute, Cary, NC, USA). A p-value of < 0.05 was assumed to indicate a statistically significant difference, except for with the SPM8 analysis. Using Spearman’s rank correlation, we assessed the relationship between the subcategory scores (core: 0–22, sleep: 0–4, activity: 0–8, psychic: 0–8, somatic anxiety: 0–6, and delusion: 0–8) of the Hamilton Depression Rating Scale (HAM-D) and the GM volume in a brain region with significant clusters obtained on the VBM analysis.

## Results

### Demographic and Clinical Data

There was no significant difference between the two groups in the distribution of sex and age (Table 1). There was a significant difference between the two groups in the total GM volume, with the volume lower in MDD patients than in HS (p < 0.05).

**Table 1:** Demographic and clinical characteristics of participants.

	HS (n = 44)	MDD (n = 27)	p
Age, mean (SD)	41.2 (11.6)	45.8 (12.7)	0.13
Female, numbers	12	12	0.14
Total gray matter volume, mean (SD) (ml)	698.7 (63.0)	665.6 (65.7)	0.04
HAMD17, mean of total scores (SD)		21.8 (4.71)	

Abbreviations: HS = healthy subjects; MDD = Major depression disorders; HAMD = 17-item Hamilton Rating Scale for Depression

Whole-brain VBM Analysis (MDD vs. HS with the s/s genotype of 5-HTTLPR).

**Figure 1:**
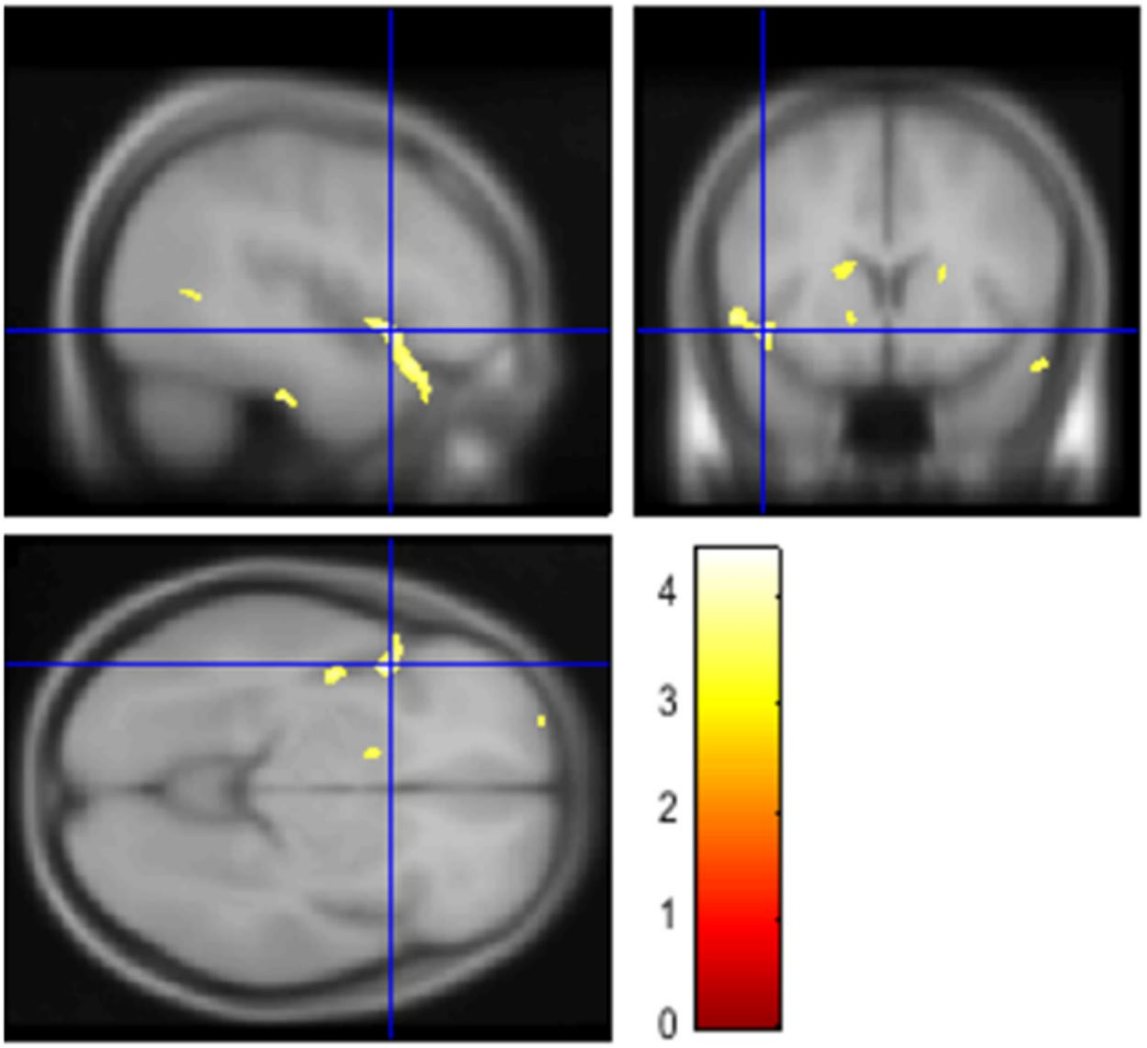
The diagnosis effects (MDD vs. HS with the s/s genotype of 5-HTTLPR). The SPM^45^ is displayed on the T1 weighted MRI. The volume of the left insula lobe was smaller in MDD patients than in HS (FWE-corrected p = 0.049, T = 4.40). The color bar represents the range of the *T*-value.

The whole-brain VBM analysis demonstrated that the volume of the left insula lobe was significantly smaller for the MDD patients than for the HS (FWE-corrected p = 0.049) (Fig. 1, Table 2). In other regions, we found no significant differences of the brain volume between the MDD patients and the HS.

**Table 2:** Results of VBM analysis.

Anatomical regions	FWE corrected p	uncorrected p	Cluster size	T-value	MNI coordinates		
	(cluster level)	(cluster level)		(Voxel level)	x	y	z
Diagnosis effects (MDD < HS)							
Left insula lobe*	0.049	0.006	2060	4.40	−44	12	−7
				4.25	−54	14	−3
				4.21	−48	24	−25

Abbreviations: FWE = family wise error rate; HS = healthy subjects; MDD = Major depression disorders.

*See Figure.

### ROI analysis of significant clusters

**Figure 2:**
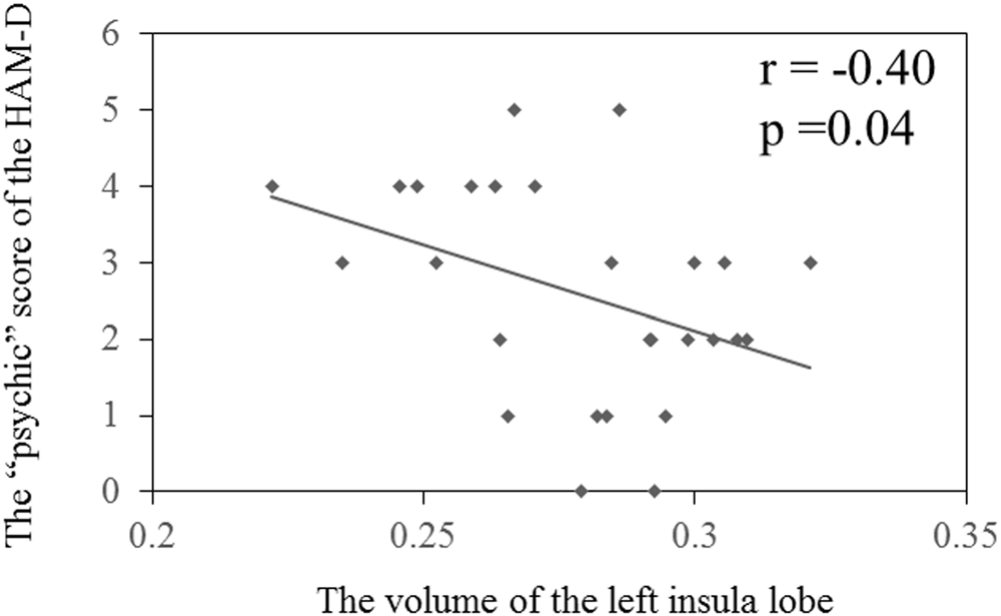
Relationship between the “psychic (items 9 and 10)” score of the HAM-D and the volume of the left insula lobe. The higher “psychic (items 9 and 10)” score of the HAM-D is associated with the volume reduction of the left insula lobe (Spearman rank correlation: uncorrected-p = 0.04, R = −0.40).

We obtained the subcategory scores of HAM-D from all 27 MDD patients. There was no relationship between total HAM-D score and the volume of the left insula lobe. We assessed relationship between the subcategory scores of HAM-D and the volume of the left insula lobe with significant clusters in the VBM analysis. Our exploratory analysis showed a significant correlation between the “psychic (items 9 and 10)” score of the HAM-D and the volume of the left insula lobe (Spearman rank correlation: uncorrected-p = 0.04, R = −0.40) (Fig. 2, Table 3). We found no relationship between other subcategory scores and the volume of the left insula lobe.

**Table 3:** Relationship between the scores of Hamilton Depression Rating Scale (HAM-D) and GM volume of the left insula lobe.

	scores (mean ± SD)	Spearman rank correlation coefficients	
		R	p
Total (0–52)	21.8 ± 4.71	−0.25	0.19
Subcategory			
core (0–22)	10.3 ± 2.80	−0.26	0.18
sleep (0–4)	3.48 ± 1.03	0.19	0.35
activity (0–8)	4.00 ± 1.22	−0.23	0.25
psychic (0–8)	2.59 ± 1.37	−0.40	0.04*
somatic anxiety (0–6)	3.67 ± 1.31	0.03	0.89
delusion (0–8)	1.59 ± 1.03	−0.10	0.63

*p < 0.05.

Ranges of each subcategory score are reported in parentheses.

**Figure 3:**
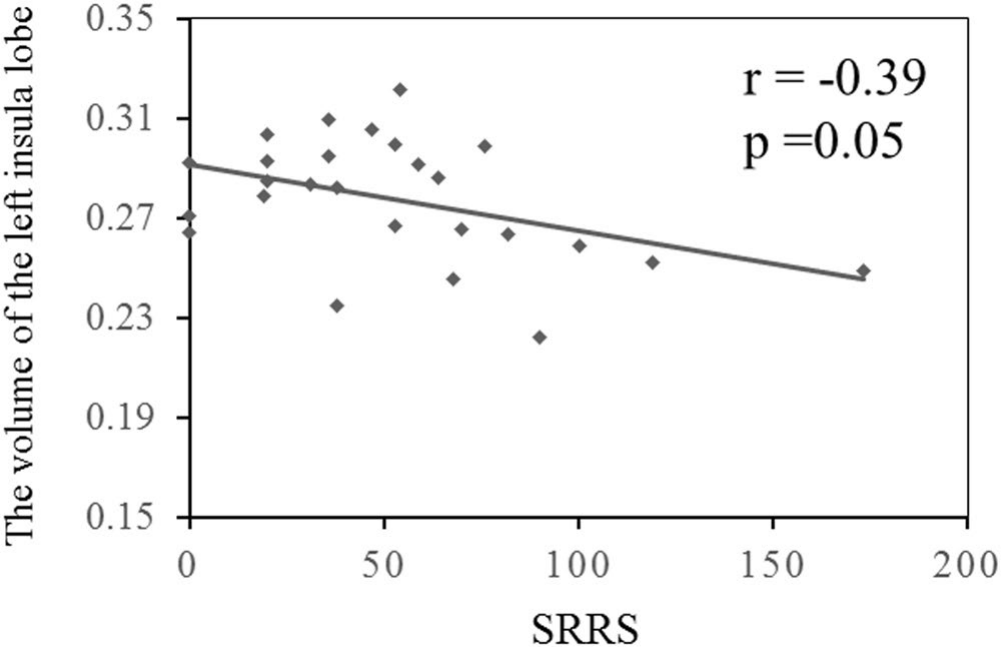
Relationship between the SRRS and the volume of the left insula lobe. The higher SRRS is associated with the volume reduction of the left insula lobe (Spearman rank correlation: uncorrected-p = 0.05, R = −0.39).

We obtained data of SRRS from 26 of 27 MDD patients. For relationship between the SRRS and the volume of the left insula lobe, an inverse tendency was observed (Spearman rank correlation: uncorrected-p = 0.05, R = −0.39) (Fig. 3).

## Discussion

Previous studies have shown difference in the allele frequency between Japanese and Caucasian populations, with a low frequency of the l-allele in Japanese populations^9^. In the current study, there were no MDD patients with the l/s or l/l genotype of 5-HTTLPR, although the reason for this predominance of s/s is unclear. Therefore, we assessed the differences in the GM volume between the MDD patients with the s/s genotype of 5-HTTLPR and the HS with the same genotype. We found that the volume of the left insula lobe was significantly smaller in the MDD patients than in the HS. Of note, there was a significant negative correlation between the volume of the left insula lobe and the “psychic” score, which consists of the sum of items 9 (agitation including mainly restlessness and psychic agitation) and 10 (psychic anxiety including apprehension, fear, panic, worry, as well as irritability) of the HAM-D, whereas other subcategory scores showed no significant correlation with the left insula lobe volume. Furthermore, the inverse tendency was observed for relationship between the volume of the left insula lobe and the SRRS. This findings strength that dimensional, but not categorical approach for MDD is reasonable. Ohira *et al*. also reported that Japanese individuals with the s/s genotype of the 5-HTTLPR showed greater blood pressure reactivity to an acute stressor than those with the s/l or l/l genotype^6^. Taken together, it is speculated that s/s individuals in Japanese are prone to become depression by psychological stress.

Frodl *et al*. examined the association between the 5-HTTLPR genotype and GM volumes in MDD patients and showed that patients with the s-allele had significantly smaller subregions within the hippocampus, dorsolateral (DLPFC) and dorsomedial prefrontal cortex (DMPFC) than HS with the same genotype^28^. However, their series for the s-allele included not only the s/s genotype but also the l/s genotype. Therefore, no previous studies have performed a simple comparison of the brain volume between MDD patients and the HS strictly regarding the s/s genotype of 5-HTTLPR. To our knowledge, this study provides the first evidence of structural brain alterations in MDD patients with the s/s genotype of 5-HTTLPR. Furthermore, the strength of this study lies in the recruitment of medication naïve MDD patients. Previous studies have shown that the alterations in the brain volume may occur during the course of MDD and after antidepressant treatments^29–31^. Therefore, the morphological changes of the insula might be related to the acute state of MDD, but not the effects caused by antidepressant treatments or the illness chronicity of MDD.

Although the previous study with the relatively large sample size of first-episode medication naïve MDD patients showed widespread GM volume reductions throughout parietal, temporal, limbic regions, and cerebellum^32^, we found only insular cortex change. This result suggests that the volume reduction of the insular lobe may occur prior to other brain regions in MDD patients with the s/s genotype of 5-HTTLPR. The insular lobe is involved in the processing of visceral sensory, visceral motor, vestibular, attention, pain, emotion, verbal, motor information, and input related to music and eating, in addition to gustatory, olfactory, visual, auditory, and tactile data^33^. Recent neuroimaging data, including VBM and fMRI, have revealed that the insular lobe was involved in MDD patients. Our findings are supported by many previous VBM studies that demonstrated a volume reduction of the insular lobe in MDD patients^34–36^; Peng *et al*. showed a volume reduction of the bilateral insular lobe in first-episode MDD patients^36^. Previous resting-state fMRI studies demonstrated that functional disconnectivity during the resting state has been observed in the insula of MDD patients. Liu *et al*. demonstrated that, compared with HS, MDD patients exhibited significantly decreased regional homogeneity, which measures local connectivity, in the right insula lobe^37^. Iwabuchi *et al*. found a failure of reciprocal influence between the insula and higher frontal regions in addition to a weakening of influences from sensory regions to the insula lobe^38^. Importantly, Yao *et al*. showed that a local connectivity reduction in the insula lobe correlated positively with anxiety^39^. This result supports our finding that there was the significant negative correlation between the volume of the insula lobe and the “psychic” score, including agitation and psychic anxiety.

Lesch *et al*.^4^ reported that the s-allele of 5-HTTLPR was related to anxiety-related traits in healthy Caucasian subjects. Gunthert *et al*.^40^ and Petersen *et al*.^41^ also observed that individuals with at least one s-allele who experienced more stressors reported more symptoms of anxiety. The current study showed the negative correlation between the SRRS and the volume of the left insula lobe. Insula lobe is thought to play a key role in mediating homeostatic responses to stress^42^. Our result also may be supported by previous study with healthy subjects which demonstrated that, compared to l-allele, the s-allele appears to be associated with greater reactivity to negative emotional stimuli in the insula^43^. Thus, we speculate that stress events might at least partially affect the insula aberrations. In other words, the integrity of the left insula lobe might be more sensitive to stressful life events than other regions in medication naïve MDD patients with the s/s genotype of the 5-HTTLPR.

Some limitations associated with the present study warrants mention: Because we assessed only subjects with the s/s genotype of 5-HTTLPR, we were unable to examine the interactions between the diagnosis and genotype. In the future, larger studies including MDD patients with the l/l genotype of 5-HTTLPR will be required to confirm our findings. All MDD patients in this study were right-handers, although roughly 10% of humans are left-handed. However, we believe that this was unlikely to affect our results, because the previous study found no significant differences in cortical volume between left- and right-handers^44^. MDD is a heterogeneous disorder. However, due to our relatively small sample size, we could not evaluate differences among various MDD subtypes that described in the previous reports^10–14^.

## Conclusion

In a Japanese population with the s/s genotype of 5-HTTLPR, we found a volume reduction of the insula in the early stages of MDD patients compared with HS. This volume reduction also showed a significant positive correlation with the “psychic” score of the HAM-D and the SRRS related to the stressful life events. Our results suggest that, in medication naïve MDD patients with the s/s genotype of the 5-HTTLPR, the insula lobe may play an important role in the “psychic” symptom and the reactivity to stress events. In the future, larger studies including MDD patients with the l/l genotype of 5-HTTLPR will be required to confirm the effect of genotype of 5-HTTLPR.
